# Polymer Stabilized Liquid Crystal Smart Window with Flexible Substrates Based on Low-Temperature Treatment of Polyamide Acid Technology

**DOI:** 10.3390/polym11111869

**Published:** 2019-11-13

**Authors:** Yang Zhang, Changrui Wang, Wei Zhao, Ming Li, Xiao Wang, Xiulan Yang, Xiaowen Hu, Dong Yuan, Weiping Yang, Yi Zhang, Pengrong Lv, Jialin He, Guofu Zhou

**Affiliations:** 1 Solar Energy Research Institute, Yunnan Normal University, Kunming 650500, China; yangzhang1114@126.com (Y.Z.); yangwpkm@126.com (W.Y.); 2 SCNU-TUE Joint Lab of Device Integrated Responsive Materials (DIRM), National Center for International Research on Green Optoelectronics, South China Normal University, No. 378, West Waihuan Road, Guangzhou Higher Education Mega Center, Guangzhou 510006, China; 2018023187@m.scnu.edu.cn (C.W.); xiaowang@m.scnu.edu.cn (X.W.); 2018023131@m.scnu.edu.cn (X.Y.); xwhu@m.scnu.edu.cn (X.H.); yuandong@scnu.edu.cn (D.Y.); zhangyi@m.scnu.edu.cn (Y.Z.); pengrong.lv@m.scnu.edu.cn (P.L.); 2018023125@m.scnu.edu.cn (J.H.); guofu.zhou@m.scnu.edu.cn (G.Z.); 3 Guangdong Provincial Key Laboratory of Optical Information Materials and Technology & Institute of Electronic Paper Displays, South China Academy of Advanced Optoelectronics, South China Normal University, Guangzhou 510006, China; 4 Shenzhen Guohua Optoelectronics Tech. Co. Ltd., Shenzhen 518110, China

**Keywords:** PSLC, smart window, flexible substrate, alignment layer, PAA

## Abstract

Polymer stabilized liquid crystal (PSLC) devices can be used as smart privacy windows that switch between transparent and opaque states. The polyimide alignment layer of a PSLC device is usually obtained by the treatment of polyamide acid (PAA) with temperatures over 200 °C. This hinders the fabrication of PSLC devices on flexible substrates, which melt at these high temperatures. In this work, the fabrication of a PSLC alignment layer using a lower temperature that is compatible with most flexible substrates, is demonstrated. It was found that the treatment of PAA at 150 °C could generate the same alignment for liquid crystals. Based on this, a PSLC device was successfully fabricated on a flexible polyethylene terephthalate (PET) substrate, demonstrating excellent electro-optic performances.

## 1. Introduction

In modern buildings, windows can be seen everywhere. The current function of a window is relatively simple. A number of recent innovations about electrically switchable windows and flexible windows have provided new properties for windows, such as privacy windows, energy-saving windows, and smart windows [1,2,3,4,5,6,7]. Among various types of electrically switchable windows, liquid-crystal-based (LC) windows have attracted great attention due to their fast response, low cost, and simple structure [8,9,10,11,12].

The development of LC windows can be mainly categorized into polymer dispersed liquid crystals (PDLCs) and polymer stabilized liquid crystals (PSLCs) [13,14]. For PDLCs, the micro- or nanometer LC droplets are dispersed in a continuous polymer matrix. Without an E-field, the LCs within the droplets are randomly oriented and the effective refractive index mismatches that of the polymer matrix, causing scattering of the incident light and the device appears opaque. When an E-field is applied, the LCs orient along the electric field, the difference in refractive index disappears, and the device turns transparent [15,16,17,18]. However, due to high polymer content in PDLCs, the working voltage is relatively high (about 100 V) [19,20]. In addition, at large viewing angles the PDLC is opaque even after the voltage is applied. 

In a PSLC there is a much lower polymer content (usually less than 10 wt%), which disperses in a continuous LC phase. A PSLC is transparent when no voltage is applied, and becomes opaque after applying a voltage [21,22,23]. Due to the low polymer content, the anchoring force between the polymer and LC is much weaker compared to a PDLC, so the working voltage is smaller (about 40 V).

Currently, E-field switchable windows typically use indium tin oxide (ITO) glass as the substrate, which cannot easily adapt to curved or specially shaped windows, thus limiting the application range of PSLC windows. Therefore, the development of a flexible PSLC film has gained great interest. For PSLC devices, the alignment layer plays a critical role in the LC orientation in the off-state and device operation mechanism. Conventionally polyimide is used as an alignment layer [24,25,26], due to its excellent chemical stability, outstanding mechanical properties, and extraordinary dielectric properties. The preparation of a functional polyimide alignment layer is usually carried out by the dehydration cyclization of the precursor polyamide acid (PAA) by thermal or chemical imidization [27,28,29]. This process usually requires a high processing temperature of over 200 °C [30,31,32]. However, common flexible substrates made of polycarbonate (PC) or polyethylene terephthalate (PET) melt at such high temperatures, which complicates the fabrication of a flexible PSLC device.

In this work, we demonstrate the fabrication of a PSLC alignment layer using relatively low temperatures. We found that treatment of PAA with lower temperatures (incompletely imidization) neither negatively impacts the inducement of homeotropic alignment of LCs, nor does it influence the formation of the polymer network in the PSLC. In this way, we successfully fabricated a PSLC device on a flexible PET substrate with a similar electro-optic performance as that of a regular non-flexible PSLC device.

## 2. Experimental Section

### 2.1. Materials

**Figure 1:**
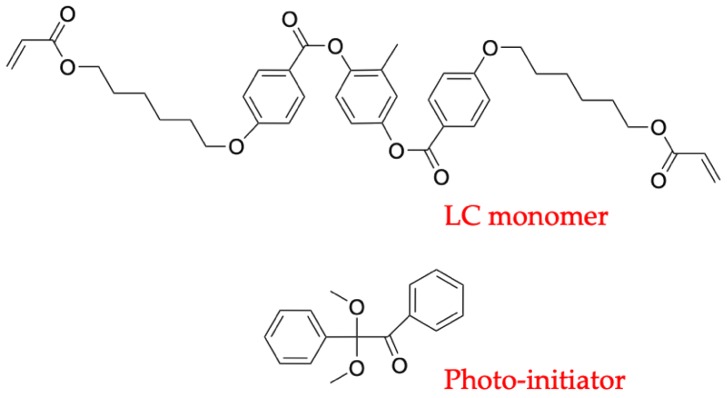
Molecular structure of the chemicals used for the fabrication of polymer stabilized liquid crystal (PSLC).

PAA solution (5 wt.% dissolved in N-methyl-2-pyrrolidone (NMP), DL-4018) was purchased from Shenzhen DALTON Electronic Material Co., Ltd (Shenzhen, China). The LC mixtures were made by mixing negative LC (T_NI_ = 94 °C, ∆ε = −8.3, ∆n = 0.149, 96.8 wt.%), LC monomer (Methylhydroquinone bis(4-(6-acryloyloxyhexyloxy) benzoic acid) ester, 3 wt.%) and photo-initiator (Irgacure 651, 0.2 wt.%) (Figure 1). The LCs were purchased from Jiangsu Hecheng Display Technology Co., Ltd. (Nanjing, China). Photo-initiator was purchased from TCI Development Co., Ltd. (Shanghai, China).

### 2.2. Methods

#### 2.2.1. Preparation of LC Cells

For the preparation of LC cells with a homeotropic alignment, ITO glass was treated by UV-ozone (BZS250GF-TC, Shenzhen HWOTECH Co., Ltd., Shenzhen, China) for 20 min. Subsequently, the ITO substrates were spin-coated with a PAA solution at 2,500 rpm for 60 s. After spin-coating, the substrates were pre-heated on a heating plate at 90 °C for 60 s, and then post-heated at 105 °C for 30 min to further remove the solvent. Finally, the substrates were heated at different temperatures (130 °C, 150 °C, 180 °C, and 230 °C) for 90 min. Among them, 230 °C is the manufacturer recommended standard processing temperature. The other three temperatures were chosen based on the DSC results and will be explained later. After cooling to room temperature, two pieces of polyimide-coated ITO glass were placed facing each other and separated by 10 μm spacers (Suzhou Nanomicro Technology Co., Ltd., Suzhou, China). The cells were filled with the LC mixtures at 60 °C. Finally, the filled cells were exposed to UV light (27 mW/cm^2^, 5 min, LingKe-1515SKA, Guangzhou Lmkoo Optoelectronic Equipment Co., Ltd., Guangzhou, China) to complete photo-polymerization.

#### 2.2.2. Measurement

Differential scanning calorimetry (DSC) was performed using a METTLER TOLEDO DSC 1 (METTLER TOLEDO, Zurich, Switzerland); scanning was performed from 105 °C to 230 °C in nitrogen and a heating rate of 2 °C/min was employed. Fourier transform infrared (FT-IR) measurement was performed on a Nicolet 6700 spectrometer (Thermo Fisher Scientific, Waltham, MA, USA). Polarized optical microscopy (POM) was performed on a Leica DM 2700P (Leica, Wetzlar, Germany). The pretilt angle was measured by a retardation measurement system RETS-100 (OTSUKA ELECTRONICS, Osaka, Japan) through the rotating analyzer method. Root mean square (RMS) surface roughness was acquired using surface profilometry system Leica DCM8 (Leica, Wetzlar, Germany). Contact angles with water were measured using a Dataphysics OCS 15 Pro (Dataphysics, Filderstadt, Germany). The transmission spectra of PSLC devices were obtained using spectrophotometer Ocean Optics Maya 2000Pro (Ocean Optics, Dunedin, FL, USA) using ITO glass as the baseline. The haze values of the devices were measured by the Haze meter CS-700 (CHN Spec, Hangzhou, China).

## 3. Results and Discussion

### 3.1. Low Temperature Treatment of PAA

The imidization conditions of PAA critically depends on its molecular structure. In order to monitor the process, DSC was firstly used to study the impact of temperature. According to the manufacturer, the as-received PAA solution contained a large quantity of NMP solvent (~95 wt.%). To ensure full evaporation of solvent in the DSC sample, and DSC signal was not interfered by solvent evaporation, and the pre-treatment (heating at 105 °C for 30 min) of PAA solution was conducted before DSC test.

**Figure 2:**
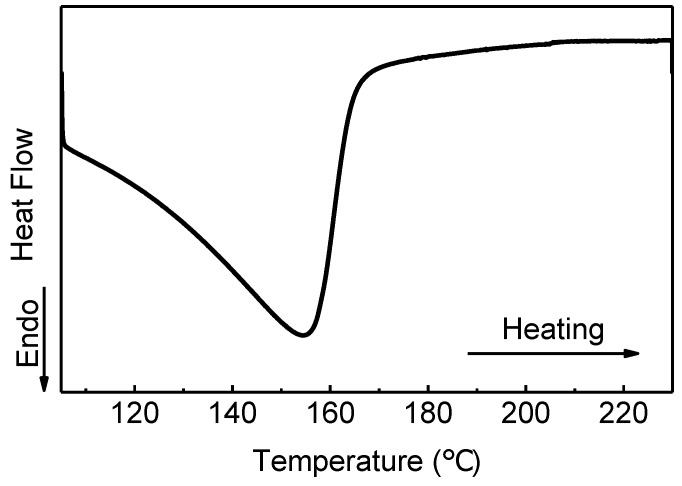
DSC of heating the pre-treated polyamide acid (PAA) sample from 105 °C to 230 °C.

As seen in Figure 2, there is a significant heat absorption peak around 155 °C, which corresponds to the reaction of dehydrating cyclization during the imidization process. According to the literature, 150 °C is also the temperature at which imidization of typical PAA has just occurred [29,33]. The DSC result suggests it is possible to initiate imidization of PAA and obtain a functional alignment layer at such low temperatures. As a result, four representative temperatures were chosen for the following experiments: 130 °C, 150 °C, 180 °C, and 230 °C.

**Figure 3:**
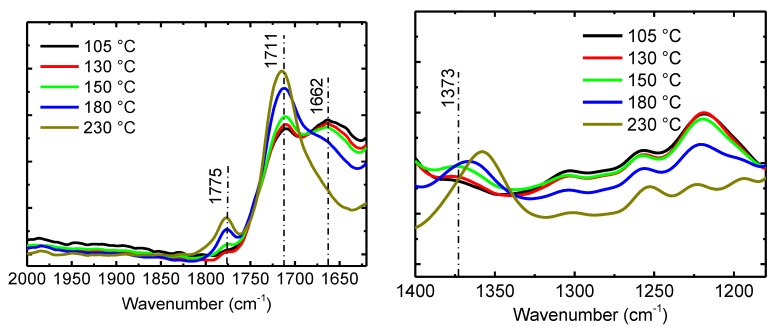
IR spectra showing pre-treated PAA and samples after the imidization process at different temperatures.

IR was next used to examine the imidization of PAA at these four temperatures (Supplementary Materials Figure S1). Figure 3 shows the IR spectra of the control pre-treated PAA and samples after the imidization process at different temperatures. The spectra of the two samples, pre-treated PAA (labeled as 105 °C) and the one with additional processing at 130 °C for 90 min, are almost the same. Combined with previous DSC result, it can be concluded that at such temperatures, an imidization reaction barely occurred. With an increase in the processing temperature, the IR spectra begin to become different, showing the signs of PAA imidization. There are three main IR peaks that can be used to follow the PAA imidization reaction. 

■ The peak at 1662 cm^−1^ was due to the stretching vibration of carbonyl group in the amide group, and its strength gradually weakened until it fully disappeared when the heating temperature increased. 

■ The peak around 1373 cm^−1^ was due to the C–N stretching vibration in polyimide, and it was enhanced as the heating temperature increased.

■ The peaks at 1711 cm^−1^ and 1775 cm^−1^ were due to the telescopic vibrations of C = O, and the strength was gradually enhanced with increasing temperature.

The observed changes suggest that the amide group of PAA disappeared completely after cyclization with the carboxyl group and polyimide forms when the sample was processed at 230 °C. When the curing temperature was between 150 °C and 230 °C, imidization can start but cannot be completed in the given time. The higher the temperature was, the higher was the degree of imidization that could be obtained.

### 3.2. PSLC with Low-Temperature Treated PAA on Glass

#### 3.2.1. Homeotropic Alignment 

**Figure 4:**
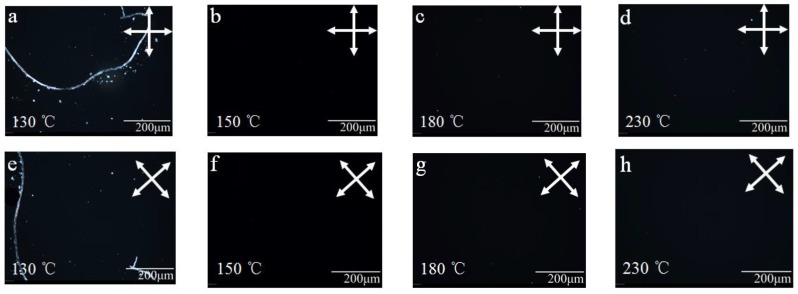
Polarized optical microscopy (POM) of liquid crystal (LC) cells with polyimide alignment layer prepared at different temperatures. (**a**) and (**e**) POM of polyimide prepared at 130 °C. (**b**) and (**f**) POM of polyimide prepared at 150 °C. (**c**) and (**g**) POM of polyimide prepared at 180 °C. (**d**) and (**h**) POM of polyimide prepared at 230 °C.

We filled a negative LC mixture into a PSLC cell and studied the alignment with polarized optical microscopy under crossed polarizers (Figure 4). The PSLC cells fabricated with the PAA heated to 150 °C or higher showed a good homeotropic alignment. However, the PSLC cell fabricated with PAA heated to 130 °C showed an alignment defect (Figure 4a,e). A processing temperature of, at least, 150 °C during the fabrication of the alignment layer was, thus, necessary to obtain a good homeotropic alignment.

To further confirm the homeotropic alignment of LCs, we measured the pretilt angle by RETS-100 (Table 1). Independent of the processing temperature, the pretilt angles of LC cell were close to 90°, which confirmed a nearly perfect homeotropic alignment in these LC cells. The imperfect alignment for polyimide layer prepared at 130 °C was presumably because the imidization of PAA had not yet started, as such the integrity of polyimide film was not ideal. This was reasonable since RETS-100 only measures a small area in the LC cell and represents the local property of the alignment layer. When processed above 150 °C, an imidization reaction occurs, and the degree of imidization increases with increasing temperature.

In addition, surface roughness and contact angles were measured in order to understand the difference observed for polyimide alignment layer prepared at different temperatures. Table 2 shows the RMS surface roughness of all samples, which were approximately 3.5 nm. Such difference in surface topography was negligible for polyimide layers prepared at all temperatures (Figure S2a–d). It was, thus, concluded that the randomly distributed topographical features of the film surface were unlikely to be the main reason for the previously observed difference of negative LC aligned on such surfaces.

**Figure 5:**
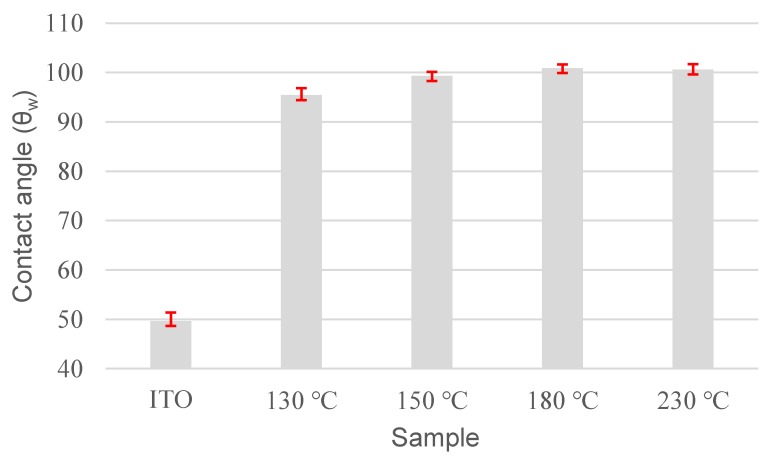
Contact angle with water on polyimide alignment layer prepared at different temperatures.

Figure 5 shows contact angles measured with water for polyimide alignment layer prepared at different temperatures. The value of the water contact angle (θ_w_) for bare ITO substrate was 49.6°. For a polyimide-coated surface processed at 130 °C, the value of θ_w_ was 95.4°. With an increase in processing temperature, the θ_w_ value became almost constant at 100°, indicating that the alkyl group of the polyimide side chain was fully stretched out and the surface became hydrophobic at this time. Such molecular conformation was presumably the main reason why the polyimide layer could induce the homeotropic alignment of LC molecules.

According to the above analysis, the low-temperature-treated PAA and partially imidized polyimide layer could provide almost identical homeotropic alignment of LCs, compared to the standard high temperature process, as long as the processing temperature was above 150 °C. Surface roughness and water contact angles indicated that the resultant polyimide films were nearly all the same from the varied processing temperatures. Overall, heat treatment of PAA between 150 °C and 230 °C could all produce a good homeotropic alignment of LCs.

#### 3.2.2. Electro-Optic Properties

**Figure 6:**
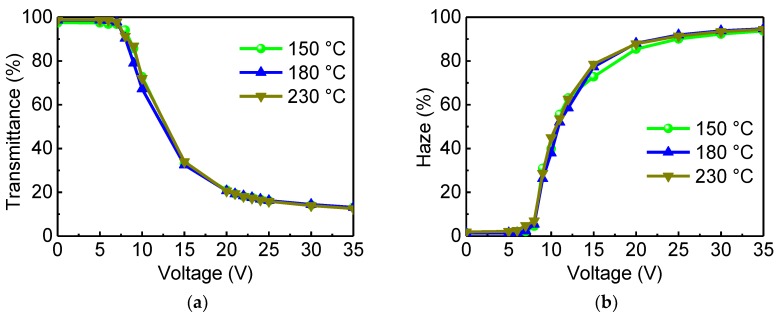
Electro-optic performance of PSLC devices. (**a**) Transmittance of the PSLC with an increase in voltage. (**b**) Haze of the PSLC with an increase in voltage.

Based on the investigation of LC device, the low temperature processing procedure was applied to the fabrication of the PSLC device. We measured the transmission at 550 nm of the PSLC device as a function of the applied voltage (Figure 6a). There was no difference for the PAA treated with different temperatures. All fabricated PSLC devices demonstrated a good electro-optic performance. With a voltage of 35 V applied, parallel light transparency of the device dropped from 98% to 13%.

The haze value of the PSLC device was also measured (Figure 6b). Different temperatures of the PAA treatment showed no effect on device performance. With applied voltage increases to 35 V, the haze of the PSLC device gradually increased to about 90%. Measured response times were almost the same for these devices as well (Table S1). In addition, we performed long-term stability experiments on fabricated PSLC devices, including high temperature (Figure S3), high humidity (Figure S4), and switching tests (Figure S5). These results showed that the PSLC devices with low-temperature-treated PAA layers have a similar stability.

### 3.3. PSLC with Low-Temperature Treated PAA on a Flexible Substrate

**Figure 7:**
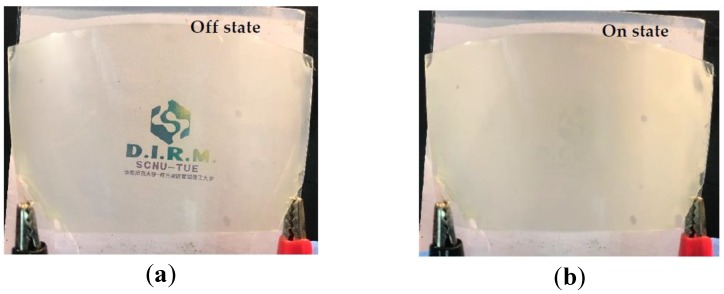

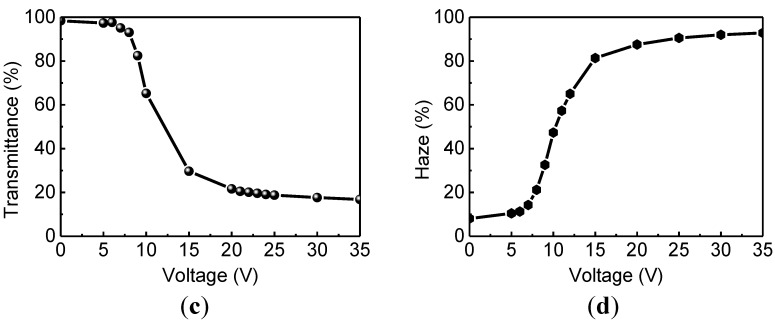
(**a**) Image of PSLC on PET substrate is off state. (**b**) Image of PSLC on PET substrate is on state. (**c**) Transmittance at 550 nm of the PSLC on PET substrate with an increase in the voltage. (**d**) Haze of the PSLC on the flexible substrate with an increase in the voltage.

We fabricated a PSLC device on a flexible substrate using PET-ITO with a PAA layer treated at 150 °C. Figure 7a,b respectively present the off- and on-state of the PSLC device. When the device was at the off-state (no voltage applied), the transmission at 550 nm was 98%, with a haze of 8% and the logo could be seen clearly. When a voltage of 35 V was applied, the transmission at 550 nm was 16% with a haze of 93% and the logo became almost invisible (Figure 7c,d). These results were similar to the electro-optic performance of PSLC devices on glass (Figure 6 and Figure S6**)**.

## 4. Conclusions

In summary, we successfully fabricated PSLC devices on a flexible substrate. We found that the fabrication of the alignment layer did not necessarily require temperatures above 200 °C, but could also be achieved using lower temperatures. We successfully processed PAA to a polyimide alignment layer on a flexible PET-ITO substrate at 150 °C, and prepared an electrically switchable PSLC device with a similar performance as that of a PSLC device on glass. We believe the current study has built up a solid foundation to realize flexible PSLC films in the future.

## Figures and Tables

**Table 1:** Pretilt angle of the LC cells with the polyimide alignment layer prepared at different temperatures.

Temperature (°C)	130	150	180	230
Pretilt angle (°)	89.5 ± 0.02	89.8 ± 0.04	89.9 ± 0.02	89.8 ± 0.02

**Table 2:** Root mean square (RMS) surface roughness with polyimide alignment layer prepared at different temperatures.

Temperature (°C)	130	150	180	230
RMS (nm)	3.8 ± 0.34	3.6 ± 0.47	3.4 ± 0.28	3.0 ± 0.45

## Supplementary Materials

The following are available online at https://www.mdpi.com/2073-4360/11/11/1869/s1, Figure S1: IR spectra showing pre-treated PAA and samples after imidization process at different temperatures. Figure S2: Surface morphology of polyimide prepared at different temperatures. Figure S3: High temperature test of the transmittance of the PSLC were prepared with PAA treated at different temperatures. Figure S4: High humidity test of the transmittance of the PSLC were prepared with PAA treated at different temperatures. Figure S5: Switching test of the PSLC were prepared with PAA treated at different temperatures. Figure S6: The transmittance of PSLC device prepared on ITO glass and PET-ITO. Table S1: Response times with polyimide alignment layer prepared at different temperatures. Video S1: PSLC device on a flexible substrate using PET-ITO with a PAA layer treated at 150 °C.
