# Supplementary material for: Polymer Stabilized Liquid Crystal Smart Window with Flexible Substrates Based on Low-Temperature Treatment of Polyamide Acid Technology
Source: Polymers (Basel). 2019 Nov 13;11(11):1869. doi: 10.3390/polym11111869 (PMC6918311; doi:10.3390/polym11111869)
Supplement: Supplementary file 1 [file polymers-11-01869-s001.zip › SI/polymers-630077-11.12 si.docx]

***Supporting Information***

**PSLC Smart Window with Flexible Substrates Based on Low Temperature Treatment of PAA Technology**

**Figure S1** shows the IR spectra of pre-treated PAA from 2000 cm^-1^ to 800 cm^-1^ with imidization process at different temperatures.

**Figure S1.** IR spectra showing pre-treated PAA and samples after imidization process at different temperatures.

**Figure S2** shows the surface morphology of polyimide prepared at different temperatures.


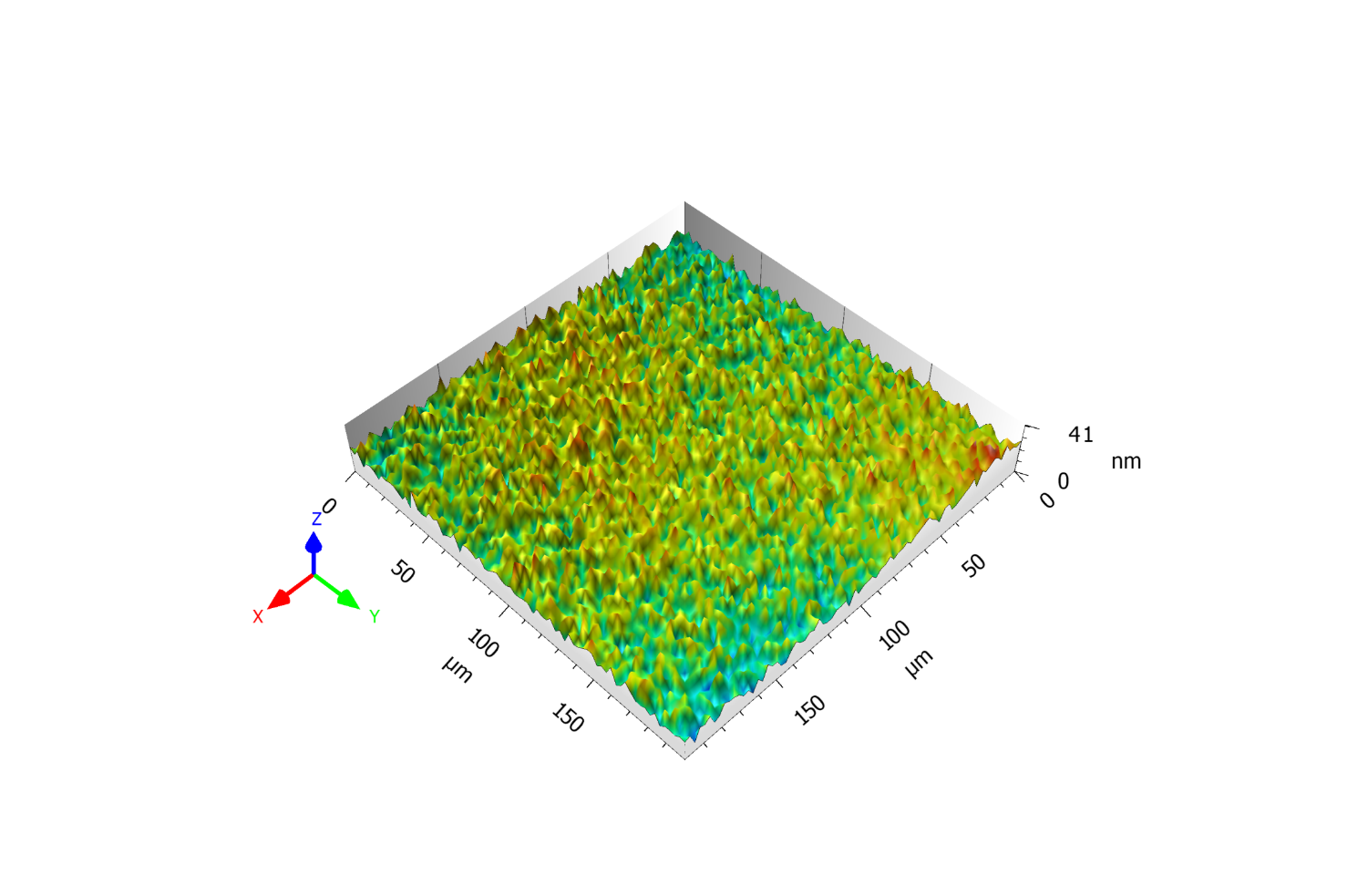

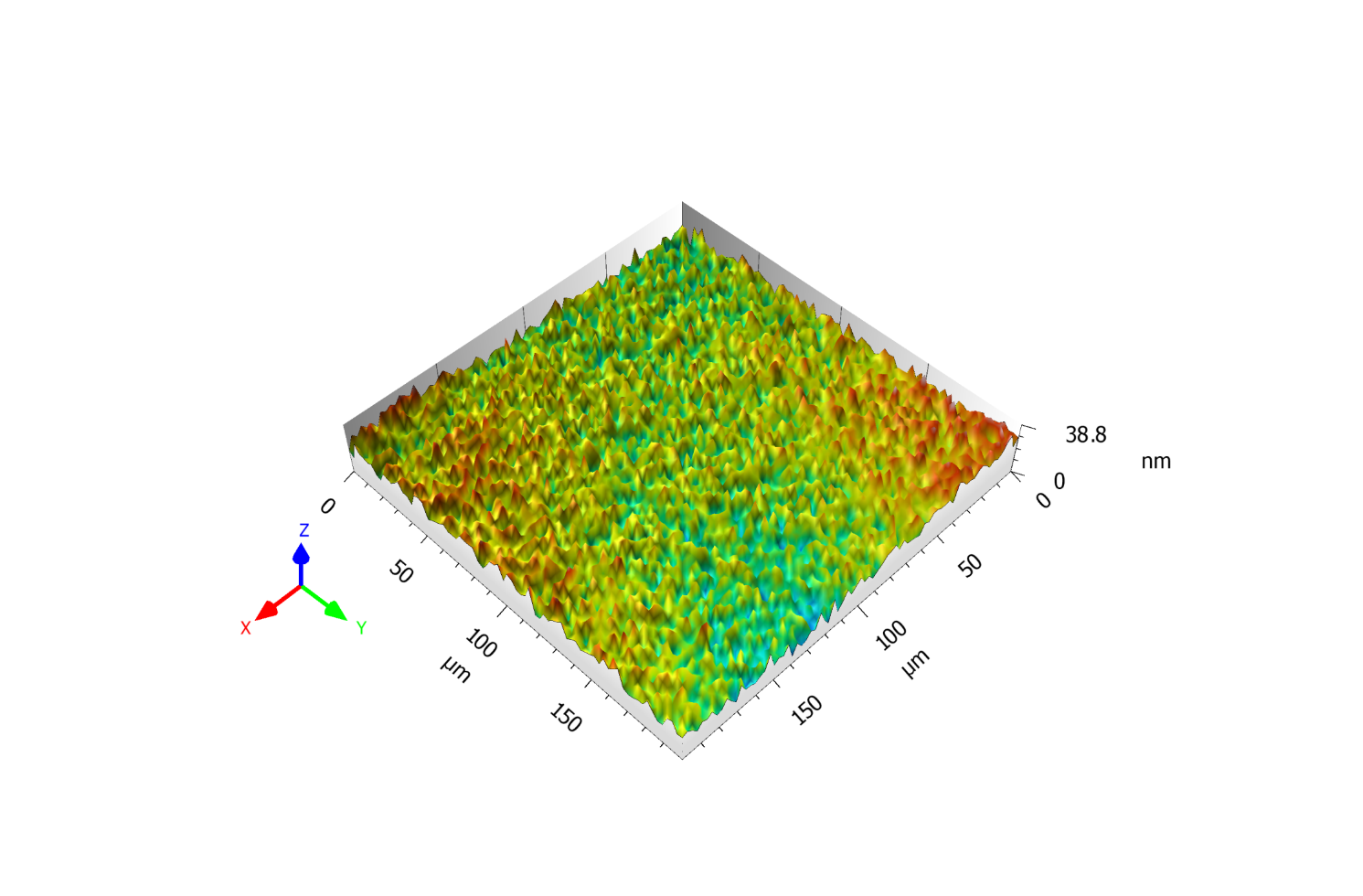

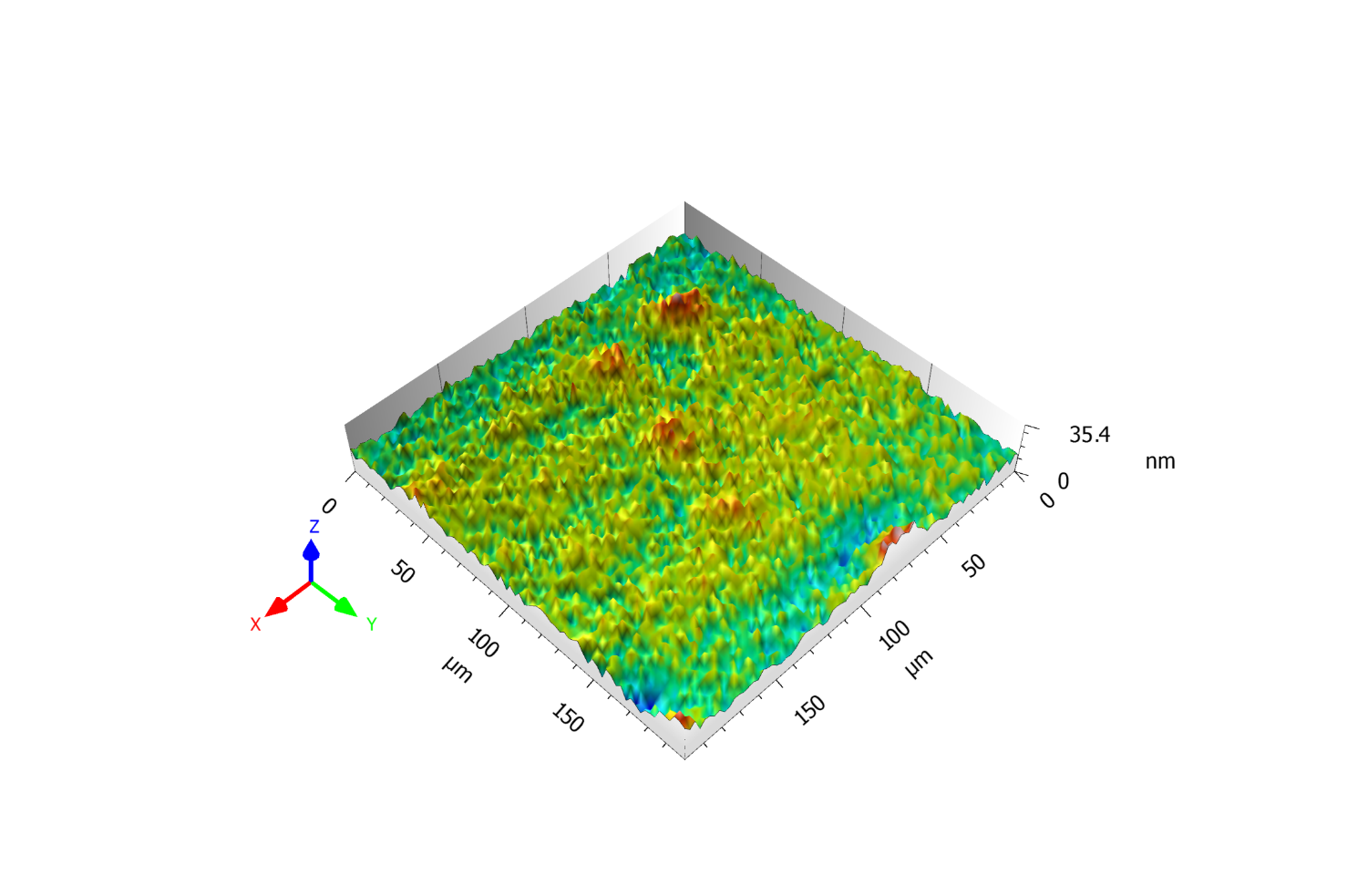

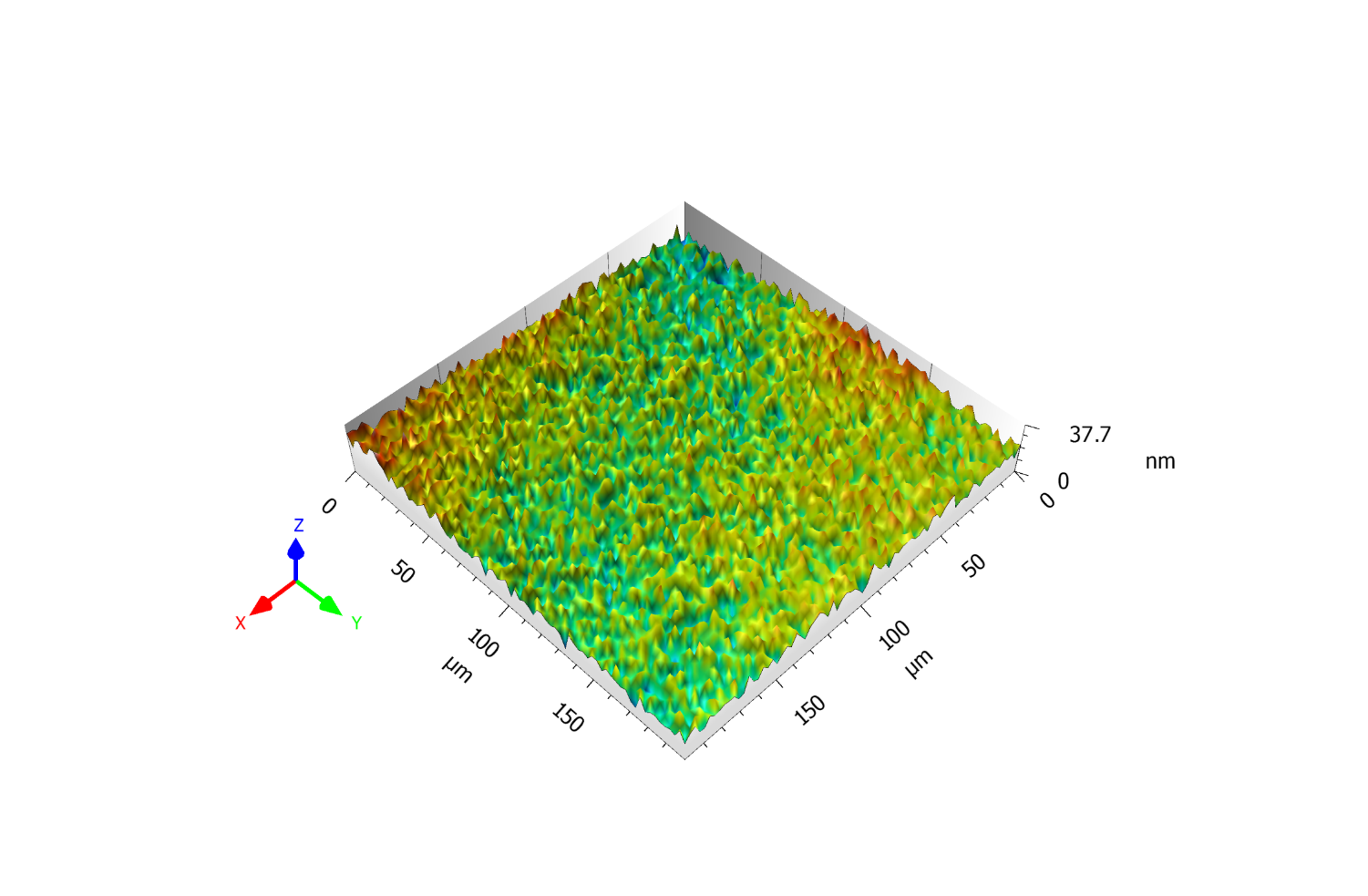


**a**

**b**

**c**

**d**

**Figure S2.** (a) Surface morphology of polyimide prepared at 130 °C. (b) Surface morphology of polyimide prepared at 150 °C. (c) Surface morphology of polyimide prepared at 180 °C. (d) Surface morphology of polyimide prepared at 230 °C.

**Figure S3** shows the results of high temperature test. The PSLC devices were prepared with PAA treated at different temperatures. Then these devices were placed in the oven at 80 °C. After 7 days later, these devices were taken out, and measure the transmittance of PSLC device at the wavelength of 550 nm as a function of applied voltage.

**Figure S3.** High temperature test of the transmittance of the PSLC were prepared with PAA treated at different temperatures.

**Figure S4** shows the results of high humidity test. Also, the PSLC devices were prepared with PAA treated at different temperatures. Place the beaker containing the saturated sodium chloride solution in the box at a room temperature of about 25 °C. When the humidity is stable at 75%, place the PSLC devices in the box and seal it. After 7 days later, take the devices out and apply the voltage.

**Figure S4.** High humidity test of the transmittance of the PSLC were prepared with PAA treated at different temperatures.

The PSLC devices were prepared with PAA treated at 150 °C and 230 °C. The sample was switched on and off every 1 second by a relay control system. Measuring the on and off states of the sample every 5000 times, as shown in **Figure S5**.

**Figure S5.** Switching test of the PSLC were prepared with PAA treated at different temperatures.

To further demonstrate that the threshold voltage of the PSLC device prepared on the flexible substrate and the ITO substrate is the same. We overlay the transmittance of PSLC devices prepared on ITO glass and PET-ITO (both treated at 150 °C) together in one plot (**Figure S6**).

**Figure S6.** The transmittance of PSLC device prepared on ITO glass and PET-ITO. For both devices PAA layers were treated at 150 °C.

We measured switching times according to the reference **S1.** The measuring device is the same as Figure 4 of reference S1. **Table S1** shows the results of response time. T_on_ means rising time (an interval from 90% transmittance to 10% transmittance); T_off_ means falling time.

**Table S1.** Response times with polyimide alignment layer prepared at different temperatures.

| Temperature (°C) | 150 | 180 | 230 |
| --- | --- | --- | --- |
| T_on_ (ms) | 1.80 | 1.98 | 1.70 |
| T_off_ (ms) | 2.46 | 2.24 | 2.50 |

**Reference**

S1 Xudong Yan; Wei Liu; Yong Zhou; Dong Yuan; Xiaowen Hu; Wei Zhao and Guofu Zhou. Improvement of Electro-Optical Properties of PSLC Devices by Silver Nanowire Doping. *Applied Science*. **2019**, *9*, 145.
